# Seizures as an Initial Manifestation of Severe Anaphylaxis Following Intrathecal Contrast Injection: A Case Report

**DOI:** 10.1155/crcc/1659767

**Published:** 2025-10-10

**Authors:** Hoa Do Thanh, Duong Le Xuan, Ghi Nguyen Hai

**Affiliations:** ^1^ Emergency Department, 108 Military Central Hospital, Hanoi, Vietnam, benhvien108.vn

## Abstract

Severe anaphylaxis is an acute allergic reaction that can be life‐threatening if not managed rapidly. Its clinical manifestations are diverse, typically including hypotension, respiratory failure, and skin manifestations. However, seizures are an uncommon presentation and may easily be overlooked. We report a case of severe anaphylaxis following intrathecal injection of a contrast agent used in the evaluation of brachial plexus injuries. The initial manifestations were seizures and hypotension, occurring 15 min after injection. The patient survived following appropriate treatment and was subsequently discharged.

## 1. Introduction

Anaphylaxis is a common medical emergency and a life‐threatening acute hypersensitivity reaction. It is defined as a rapidly developing systemic allergic reaction involving multiple organ systems. Without timely treatment, it can progress to respiratory and circulatory collapse and death. Global epidemiological data estimate that the lifetime prevalence of anaphylaxis ranges from approximately 0.05% to 2% (0.05%–2% in the United States and up to ~3% in Europe) [1]. The annual incidence has been reported at 50–112 episodes per 100,000 person‐years [2]. Additionally, recent meta‐analyses indicate a current global incidence estimate of approximately 46 cases per 100,000 population per year, with an observed annual increase of approximately 7.4% [3]. Anaphylaxis can occur at any age and in any setting. Unfortunately, it is often misdiagnosed or unrecognized at onset, leading to treatment delays and a higher risk of morbidity and mortality. Therefore, anaphylaxis remains a critical concern across all medical specialties, as it can arise unexpectedly in any clinical context.

Iodinated contrast agents are known triggers of hypersensitivity reactions, including anaphylaxis. These reactions predominantly occur via non‐IgE–mediated mechanisms, often referred to as nonallergic (pseudoallergic) reactions, involving direct mast cell and basophil activation, complement activation, and osmotic effects on cellular membranes [4]. Intrathecal contrast administration, performed for diagnostic evaluations such as brachial plexus injury assessment, involves injecting agents into the subarachnoid space after lumbar puncture. This route may provoke atypical presentations compared to intravenous administration, potentially manifesting with neurological symptoms such as seizures, in addition to hypotension [5]. The overall incidence of immediate hypersensitivity reactions to nonionic iodinated contrast media is low. Estimates indicate frequencies between 0.3% and 1.4% of administrations, with severe reactions (Anaphylaxis Grade 3) occurring in approximately 0.02%–0.04% of cases [6].

We present a clinical case of severe anaphylaxis triggered by intrathecal contrast administration that was successfully managed.

## 2. Case Report

A 20‐year‐old man with the body weight of 60 kg, sustained a brachial plexus injury following a work accident 1 year prior, which required metal fixation in his arm. On presentation, the patient was alert with a Glasgow Coma Scale (GCS) score of 15, afebrile, heart rate of 60 bpm, blood pressure 120/80 mmHg, respiratory rate of 16 breaths per minute, and SpO_2_ 96%. Complete paralysis of the right arm was noted as a sequela of the prior injury.

To evaluate the brachial plexus lesion, the orthopedic specialist requested a cervical spine CT with intrathecal contrast. Following lumbar puncture, 5 mL of Omnipaque 300 mg/mL was administered intrathecally.

Fifteen minutes postinjection, the patient developed generalized seizures, decreased consciousness (GCS 13), tachycardia (120 bpm), hypertension (160/100 mmHg), tachypnea (26 breaths per minute), and hypoxemia (SpO_2_ 92%), without urinary incontinence (Table 1). Immediate treatment in the CT suite included intramuscular adrenaline 0.5 mg, intravenous methylprednisolone 40 mg, after which the patient was transferred to the emergency department within 5 min of symptom onset.

**Table 1:** Clinical symptoms and adrenaline dosing timeline.

**Time (postinjection)**	**Heart rate**	**Blood pressure**	**NaCl 0.9% (mL)**	**Adrenaline dose (mcg/kg/min)**	**Urine output (mL/kg/h)**
15 min	120	160/100		IM 0.05 mg	
20 min	125	150/95	200	0.05	
30 min	100	120/80	500	0.1	Inserted catheter
90 min	105	115/70	800	0.28	1.5
180 min	105	90/60	1000	0.33	1.8
360 min	100	100/60	2000	0.05	2.0
24 h	98	110/70	3000	0.03	Average: 1.8 mL/kg/h
36 h	90	120/80	1000 mL/12 h	0	Average: 2.0 mL/kg/h
48 h	80	125/85	1000 mL/12 h	0	Average: 2.1 mL/kg/h
Day 3	85	125/80		0	Average: 2.2 mL/kg/h
Day 4	80	120/80		0	Average: 2.2 mL/kg/h

In the emergency department, the patient continued to experience seizures and deteriorating consciousness, with heart rate 125 bpm, blood pressure 150/95 mmHg, respiratory rate 28 breaths per minute, SpO_2_ 90%, and urinary incontinence. No erythema or bronchospasm was observed. Management included head elevation, continuous intravenous adrenaline infusion (0.05 mcg/kg/min), intravenous diazepam 20 mg, and isotonic saline (0.9% sodium chloride) at 10 mL/min. Endotracheal intubation and mechanical ventilation were initiated. Arterial blood gas (ABG) analysis revealed severe mixed acid–base disturbance with profound acidemia (pH 6.80), marked hypercapnia (pCO_2_ 81 mmHg), significant metabolic derangements including hypernatremia, hyperkalemia, hypercalcemia, hyperglycemia, severe lactic acidosis, and elevated hematocrit indicating hemoconcentration (Table 2).

**Table 2:** Arterial blood gas and lactate timeline.

**Parameter**	**Ref. range**	**Time (postinjection)**					
		**15 min**	**90 min**	**180 min**	**360 min**	**24 h**	**48 h**
pH	7.35–7.45	**6.80 ↓**	**6.80 ↓**	**6.99 ↓**	**7.14 ↓**	**7.26 ↓**	7.38
pCO_2_ (mmHg)	35–48	**81 ↑**	**115 ↑**	40	44	**32 ↓**	36
pO_2_ (mmHg)	83–108	**186 ↑**	**121 ↑**	**125 ↑**	**144 ↑**	**150 ↑**	**148 ↑**
Na^+^ (mmol/L)	136–145	**156 ↑**	**156 ↑**	**148 ↑**	144	**153 ↑**	142
K^+^ (mmol/L)	3.4–4.5	**5.0 ↑**	**4.8 ↑**	**5.6 ↑**	**5.7 ↑**	**5.0 ↑**	**4.8 ↑**
Ca^2+^ (mmol/L)	0.9–1.3	**1.48 ↑**	**1.51 ↑**	1.22	1.22	1.06	1.15
Glucose (mmol/L)	3.9–6.4	**12.0 ↑**	**11.2 ↑**	**14.0 ↑**	**12.4 ↑**	**11.1 ↑**	**9.6 ↑**
Lactate (mmol/L)	0.5–2.2	**11.2 ↑**	**25.9 ↑↑**	**16.0 ↑**	**10.6 ↑**	**3.6 ↑**	**3.6 ↑**
Hct (%)	35–51	**54 ↑**	44	38	40	38	37
HCO_3_^−^ (mmol/L)	18–23	—	—	**9.1 ↓**	**15.0 ↓**	**15.5 ↓**	21.5
Base excess (mmol/L)	−2 to +3	—	—	**−22.1 ↓**	**−14.0 ↓**	**−12.7 ↓**	+4.1 ↑
SO_2_ (%)	95–97	—	**89 ↓**	97	**100 ↑**	**99 ↑**	**99 ↑**

*Note:* Bold values indicate abnormal results outside the reference range. Arrows (↑, ↓) denote increases or decreases compared with the reference range.

Within 15 min, seizure activity diminished, and by 30 min, it had completely resolved. Vital signs stabilized with heart rate 100 bpm and blood pressure 120/80 mmHg. Mechanical ventilation was set in assist‐control mode with FiO_2_ 50%, respiratory rate 14 breaths per minute, tidal volume 450 mL, and SpO_2_ 96%. However, the adrenaline infusion required escalation to 0.1 mcg/kg/min to maintain hemodynamic stability.

Despite ongoing infusion, blood pressure continued to decline, necessitating further dose increases. Three hours later, the heart rate was 105 bpm, blood pressure 90/60 mmHg, and adrenaline was titrated up to 0.33 mcg/kg/min. A total of 1500 mL of 0.9% sodium chloride was infused, with 1000‐mL urine output over 3 h. Renal function tests remained normal. ABG analysis (Table 2) showed severe metabolic acidosis with inadequate respiratory compensation, persistent hypernatremia, hyperkalemia, hyperglycemia, and elevated lactate consistent with severe lactic acidosis, while hematocrit normalized.

After 6 h, the adrenaline dose was gradually reduced, and by 12 h, it had been decreased to 0.05 mcg/kg/min. ABG parameters showed partial improvement with higher pH, better bicarbonate and base excess values, and lower lactate levels, although significant metabolic acidosis persisted (Table 2).

At 36 h, adrenaline infusion was discontinued, with stable vital signs (heart rate 90 bpm, blood pressure 125/85 mmHg). By 48 h, the patient was successfully extubated. ABG analysis demonstrated near‐normalization, including normal pH, adequate pCO_2_, improved bicarbonate and base excess, and high oxygenation due to supplemental oxygen. Mild metabolic abnormalities persisted, such as slightly elevated potassium, glucose, and lactate, but other parameters were within normal limits (Table 2).

The patient was discharged on Day 4 with stable vital signs: heart rate 80 bpm, blood pressure 120/80 mmHg, respiratory rate 16 breaths per minute, SpO_2_ 96%, and GCS 15.

## 3. Discussion

This case illustrates a rare initial presentation of anaphylaxis in which seizures occurred before hypotension, a phenomenon reported in only a small proportion of cases (e.g., loss of consciousness in ~13% and incontinence in ~1.5% of anaphylactic episodes) [7]. Although blood pressure was initially preserved, the prompt administration of intramuscular epinephrine was appropriate and lifesaving, consistent with clinical guidelines identifying epinephrine as the first‐line therapy even when the diagnosis is not yet confirmed [8]. As the condition progressed to hypotension, escalation to intravenous epinephrine was required, while early airway protection via endotracheal intubation remained essential due to ongoing seizures and loss of airway reflexes.

At symptom onset, the differential diagnosis included primary seizure disorder, contrast‐induced neurotoxicity, and anaphylaxis. The occurrence of severe seizures as the initial manifestation, while blood pressure was still preserved, presented a major diagnostic challenge. Contrast‐induced neurotoxicity is typically suspected when patients develop root pain, paresthesia, or seizures shortly after intrathecal administration, whereas primary seizure disorders may also present with generalized tonic–clonic convulsions of similar appearance. However, the subsequent rapid deterioration with hypotension, along with the prompt and favorable response to adrenaline, ultimately supported the diagnosis of anaphylaxis. This case highlights that even in the absence of early hypotension, anaphylaxis should remain a critical differential diagnosis when acute neurological symptoms arise immediately following exposure to a potential trigger [8].

Seizures following intrathecal contrast injection have been documented. For example, accidental intrathecal administration of ionic contrast (iothalamate meglumine) resulted in ascending tonic–clonic seizure syndrome and poses a high risk of mortality [9]. Other case reports describe contrast‐induced encephalopathy characterized by seizures and focal deficits such as hemiplegia [10]. The pathophysiology of contrast‐induced neurotoxicity remains multifactorial. Mechanisms include blood–brain barrier disruption and direct neurotoxicity following agent diffusion into neural tissues, as well as hyperosmolar shifts that disrupt ionic balance and synaptic transmission [11]. Notably, nonionic contrast agents have also been implicated; several cases of convulsive status epilepticus have been reported after intrathecal administration of nonionic contrast agents, highlighting that seizure risk is not confined to ionic agents [12].

With regard to management, because intrathecal contrast is denser than cerebrospinal fluid (CSF), patients should be positioned with the head and torso elevated to limit cephalad migration. Early airway protection with endotracheal intubation and mechanical ventilation, neuromuscular paralysis, and aggressive seizure control are recommended. CSF drainage/lavage may be considered in severe cases. Adjunctive measures include aggressive IV fluids and corticosteroids, which have been used to shorten exposure and mitigate neurotoxicity. Seizure control is critical to reduce cerebral metabolic demand and excitatory neurotransmission [13, 14].

The proposed use of high‐dose methylprednisolone in contrast‐induced neurotoxicity is extrapolated from protocols established in acute spinal cord injury (NASCIS II) [15]. To date, no evidence‐based or validated therapeutic regimen exists specifically for this condition, and current recommendations rely primarily on pathophysiologic rationale and isolated case reports [16]. The literature on contrast‐induced neurotoxicity remains limited to case reports, small case series, and narrative reviews; thus, treatment strategies should be considered empirical and tailored to individual patient circumstances.

## 4. Conclusion

Seizures may represent an early manifestation of anaphylaxis following intrathecal contrast injection, even before hypotension develops. This case emphasizes the importance of early recognition, rapid administration of adrenaline, timely airway protection, and seizure control as critical measures for survival. Vigilant monitoring and consideration of anaphylaxis in the differential diagnosis of acute neurological symptoms after contrast exposure are essential for improving patient outcomes.

## Ethics Statement

The study was approved by the 108 Military Center Hospital ethics committee in biomedical research.

## Disclosure

All authors read and approved the final version of the manuscript.

## Conflicts of Interest

The authors declare no conflicts of interest.

## Author Contributions

All authors listed have significantly contributed to the investigation, development, and writing of this article. Conceptualization: Duong Le Xuan, Hoa Do Thanh, and Ghi Nguyen Hai. Data curation: Duong Le Xuan. Formal analysis: Duong Le Xuan, Hoa Do Thanh, and Ghi Nguyen Hai. Supervision: Duong Le Xuan. Writing—original draft: Ghi Nguyen Hai. Writing—review and editing: Duong Le Xuan, Hoa Do Thanh, and Ghi Nguyen Hai.

## Funding

No funding was received for this manuscript.

## Data Availability

Data will be made available on request.
